# Surgical Treatment of Intra- and Juxtamedullary Spinal Cord Tumors: A Population Based Observational Cohort Study

**DOI:** 10.3389/fneur.2019.00814

**Published:** 2019-07-26

**Authors:** Oscar Persson, Alexander Fletcher-Sandersjöö, Gustav Burström, Erik Edström, Adrian Elmi-Terander

**Affiliations:** ^1^Department of Neurosurgery, Karolinska University Hospital, Stockholm, Sweden; ^2^Department of Clinical Neuroscience, Karolinska Institutet, Stockholm, Sweden

**Keywords:** intramedullary spinal cord tumors, demographics, treatment strategies, surgical treatment, functional outcome, radiotherapy

## Abstract

**Objective:** Intramedullary spinal cord tumors (IMSCT) are rare entities and high-level evidence regarding optimal treatment is lacking. We aim to describe the demographics, histopathological distribution, onset symptoms, treatment strategies, and functional outcome for patients surgically treated for IMSCT.

**Methods:** We performed a retrospective review of a consecutive population-based cohort of 95 patients who underwent surgery for intra- or juxtamedullary tumors at a single institution during the period 2004–2017.

**Results:** When gross total resection (GTR) was achieved, we found no case of local tumor recurrence even in the absence of adjuvant radio- or chemotherapy. Meanwhile, we found a 50% progression rate on long-term MRI follow-up in patients where only a partial resection was possible. At long-term follow-up, there was no significant alteration in functional status, while a significant reduction in share of patients reporting pain, compared to preoperative status, was identified. Poor preoperative functional status and postoperative tumor remnant were identified as individual risk factors for further functional decline.

**Conclusion:** Gross total resection, with minimal post-operative neurological deterioration, is possible in the majority of the cases, especially in the presence of an identifiable resection plane between tumor and healthy spinal cord. Since long-term progression-free survival could be achieved by GTR without additional adjuvant treatment, we emphasize that low-grade tumors should not be subject to radiotherapy. Treatment of high-grade or diffusely infiltrating tumors, tumor remnants, or metastases should be individualized.

## Introduction

Intramedullary spinal cord tumors (IMSCT) are rare entities. They account for 5–10% of all spinal tumors in adults; and only about 3% of all CNS tumors (1–3). Because of the rarity of these tumors and the diversity of histological subtypes there is a paucity of high-level evidence regarding optimal treatment.

The most common histopathological entity in adults are ependymomas with an incidence of about 0.2/100,000, followed by astrocytic tumors—pilocytic or diffuse—and hemangioblastomas (4). Meanwhile, astrocytomas are the most common IMSCT in the pediatric population (2, 5, 6). Besides these, a wide variety of less common entities such as gangliogliomas, subependymomas, neurocytomas, lipomas, and PNETs accounts for the rest of the primary IMSCT.

Ependymomas arise throughout life but are most common in young or middle-aged adults. Males and females are equally affected. Although not encapsulated these tumors often have a well-defined resection plane without diffuse infiltration of surrounding tissue (7), making radical surgical resection possible. They most frequently arise in the cervical part of the spinal cord (8), and are commonly WHO grade II, although less commonly subependymomas (WHO grade I) or anaplastic ependymomas (WHO grade III) are encountered. Myxopapillary ependymomas are benign tumors (WHO grade I) commonly located in the filum terminale or at the conus region but are generally considered extramedullary.

Hemangioblastomas are richly vascularized benign tumors. They have been reported to constitute between 2 and 8% of all IMSCTs (1). The majority are sporadic, but up to 20–45% of spinal hemangioblastomas are associated with Von Hippel-Lindau (VHL) disease (9–11). They are usually small and rarely extend beyond one or two segments (1), have a non-infiltrative growth pattern with a clear tumor-parenchyma demarcation (10), and an associated syrinx is common (12).

Since ependymomas and hemangioblastomas usually have a clear resection plane, and radical excision is considered curative, surgical resection is usually the treatment of choice for these tumors (13).

Astrocytic tumors of the spinal cord are histologically heterogeneous and have a propensity to arise in the cervical or cervicothoracic region. The pilocytic astrocytomas (WHO grade I) are benign encapsulated lesions that can be cured by complete surgical excision (14). Diffuse fibrillary astrocytomas are infiltrative non-encapsulated tumors. In the spine these tumors are more commonly low grade (WHO grade II) compared to their intracranial counterpart. These low-grade tumors sometimes have a pseudocapsule, making them more amenable to radical resection. High grade anaplastic astrocytomas or GBMs are associated with older age and offer a poor prognosis without any known cure available (14). Extensive resection of these tumors is controversial, since the diffuse and infiltrative growth pattern often makes radical resection impossible without severe neurological deficits. Depending on growth pattern, appearance of resection plane, and histopathologic grade, astrocytomas could be treated conservatively, surgically, or by means of radiation and/or chemotherapy (15).

Metastatic spread to the spinal cord is very rare in surgical series and usually reported to account for <2–5% of the cases, with lung and breast cancer being the most frequent origin (16–20). Survival is generally <6 months (19), and most patients are treated non-surgically with either radiotherapy and/or chemotherapy (19, 21).

The most common onset symptom for IMSCT is diffuse non-specific axial pain. Other common onset symptoms are slowly progressing motor or sensory deficits (6). Bladder or gastrointestinal dysfunction occurs less frequently and usually later during symptom development (1, 17). Symptoms can sometimes develop slowly over years before diagnosis.

Surgery is generally considered first line treatment for most IMSCT, and radiotherapy is usually reserved for patients with malignant tumors (WHO grade III-IV) (7, 8). Intraoperative neurophysiological monitoring (IONM) using somatosensory-evoked potentials (SEP), motor-evoked potentials (MEP) and D-wave have been shown to decrease the incidence of postoperative functional neurological decline (7, 22–25). The most common surgical complication is postoperative CSF leak which occurs in about 5–10% of surgeries (12, 14).

We here present a population-based cohort study of juxta- and intramedullary tumors. The objective of the study is to describe the cohort and our experiences from it. We look to compare our findings to published data on the demographics, histopathological distribution, onset symptoms, treatment strategies, and functional outcome of these patients.

## Materials and Methods

### Patient Selection

The study was performed according to approval of the Regional Ethical Review Board, Stockholm, Sweden (2016/1708-31/4). All adult patients (16 years or older) surgically treated at the Karolinska University Hospital (Stockholm, Sweden) for intraspinal tumors during the period 2004–2017 were included in the review and all patients with intra- or juxtamedullary tumor location were included in the analysis. The follow up period was extended to obtain a minimum of 12 months of follow up for all included patients. Patients were identified through the hospital's surgical management software, Orbit (Evry Healthcare Systems, Solna, Sweden). The material was cross-referenced with the national cancer registry regarding the primary tumors to verify that no patients were missed.

### Surgical Technique

All surgeries were performed by either of three neurosurgeons as the primary attending surgeon.

Prior to surgery, the spinous process of the vertebra above the tumor (if thoracic or lumbar) was identified using CT guidance and marked with injection of a sterile carbon suspension. With the patient in the prone position, a posterior midline approach was used. Laminotomy was performed, in the majority of cases, using an ultrasonic bone scalpel (Misonix Inc., Farmingdale, New York, USA).

Ultrasound registration was performed before opening of the dura. Under the microscope, the dura was incised in midline and was held open by sutures. The arachnoid was dissected sharply, and the tumor area was exposed.

For strict intramedullary tumors, the midline was identified either visually, or in uncertain cases by using a neurophysiological grid or by dorsal column mapping (DCM). A midline myelotomy, corresponding to the craniocaudal extension of the tumor, was performed using a diamond knife. Using 6.0 Prolene sutures, the pia mater was sutured to the dura to gently keep the myelotomy open. A small number of tumors located close to the lateral surface of the spinal cord were approached via a dorsal root entry zone myelotomy lateral to the dorsal columns. The tumor was dissected sharply when possible or removed by ultrasonic aspirator (Sonopet, Stryker). When visual GTR was achieved, the myelotomy was closed and sutured using 6.0 Prolene sutures.

For juxtamedullary tumors, the cranial and caudal poles were identified, and the tumor was dissected from nerves, conus medullaris, or the surface of spinal cord depending on tumor type and location.

In all cases, watertight dural closure was performed and in most cases the lamina reinstated using microplates. The surgical wound was closed according to routine.

Intraoperative neurophysiological monitoring (IONM) was applied for strict intramedullary and some juxtamedullary tumors when technically available. Somatosensory-evoked potentials (SEP) were continuously monitored, and D-wave registration performed where possible, while motor-evoked potentials (MEP) monitoring was performed intermittently. The neurophysiological parameters were used to provide feedback during the surgery. In cases where a significant loss of signal was identified, a strategic decision was made by the surgeon regarding continuing or discontinuing the tumor resection.

### Statistical Analysis

For descriptive purposes, continuous data are presented as medians (range) and categorical data as numbers (proportion). A simple logistic regression was used to assess predictors of decreased functional outcome (measured by mMCs) following tumor resection. Following this, a forced-entry multiple logistic regression including demographic data and factors indicating a significant trend (*p* < 0.1) in the simple logistic regression, was performed to determine independent risk factors for decreased functional outcome. Permanent loss of MEP/SEP was included in the risk factor analysis (simple logistic regression) but due to data missing in 50% of cases, it was not included in the multiple logistic regression. Instead, a separate regression analysis was performed correlating IONM to functional outcome in patients with intramedullary tumors. The statistical significance level was set to *p* < 0.05. The statistical program SPSS (IBM SPSS Statistics for Macintosh, Version 25) was used.

## Results

### Patient Data

**Figure 1:**
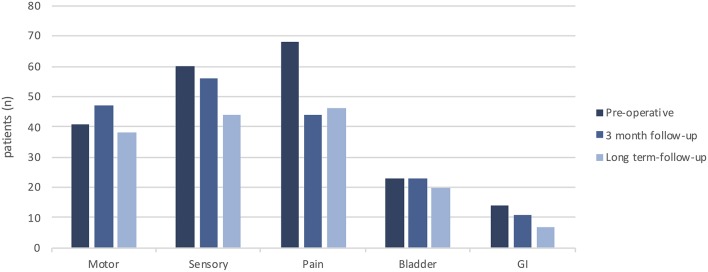
Distribution of symptoms preoperatively, at short term follow-up (3 months) and at long term follow up.

**Figure 2:**
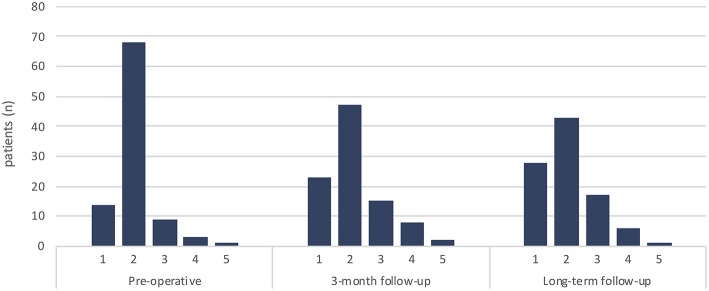
Distribution of functional status according to modified McCormic scale (mMCs) preoperatively, at short term follow-up (3 months) and at long term follow up.

In total, 95 patients were included in the study. There were 56 males and 39 females, with the median age at the time of surgery being 45 years (16–64). The median time elapsed between the onset of symptoms and surgery was 12 months (0–372). The most common symptoms were pain (72%, *n* = 68), sensory deficit (63%, *n* = 60) and motor deficit (43%, *n* = 41) (Figure 1). Two patients had previously been subjected to spinal surgery, and two had previously been treated with chemotherapy (Table 1). The median pre-operative American Spinal Injury Association Impairment Scale (ASIA IS) was D (B–E), and the median modified McCormick clinical–functional scale (mMCs) was 2 (1–5) (Table 1, Figure 2).

**Table 1:** Patient data.

**Variable**	**Entire cohort (*n* = 95)**
Male gender	56 (59%)
Age (years)	45 (16–64)
Pre-operative symptoms	Motor deficit: 41 (43%) Sensory deficit: 60 (63%) Pain: 68 (72%) Decreased bladder function: 23 (24%) Decreased gastrointestinal function: 14 (15%)
Pre-operative symptom duration (months)	12 (0–372), (3 missing, *n* = 92)
Pre-operative spinal surgery	2 (2%)
Pre-operative spinal radiation	0 (0%)
Pre-operative chemotherapy	2 (2%)
Pre-operative mMCs	2 (1–5)
Pre-operative ASIA IS	D (B–E)

*mMCs, modified McCormick scale; ASIA IS, American Spinal Injury Association impairment scale. Variables are presented as count (%) or median (range)*.

### Tumor Characteristics

The most common tumor location was the lumbar spine (Table 2). However, after exclusion of myxopapillary ependymomas (*n* = 27) which were all located in the conus region, the most common level for the remaining tumors (*n* = 68) was cervical (47%) followed by thoracic (32%). Based on the pre-operative magnetic resonance imaging (MRI), contrast enhancement was apparent in 95% of tumors (*n* = 90), and peritumoral edema in 44% (*n* = 42). Fifty-three percent (*n* = 50) of the tumors were intramedullary. Thirty six percent (*n* = 34) of tumors had a cystic component and 21% (*n* = 20) had tumor-associated syringomyelia (Table 3).

**Table 2:** Intraoperative data.

**Variable**	**Entire cohort (*n* = 95)**
Spinal level	Cervical: 32 (34%) Thoracic: 25 (26%) Lumbar: 38 (40%)
Grade of resection (postoperative MRI)	Gross total: 77 (81%) Partial: 14 (16%) Biopsy: 4 (4%)
Intramedullary	50 (53%)
Neurophysiological monitoring	49 (52%)
Transient loss of SEP	4 (8%)
Permanent loss of SEP	24 (49%)
Transient loss of MEP	2 (4%)
Permanent loss of MEP	12 (24%)
Postoperative radiation	10 (11%)
Postoperative chemotherapy	6 (6%)

*SEP, somatosensory evoked potentials; MEP, motor evoked potentials. Variables are presented as count (%)*.

**Table 3:** Histopathological diagnosis, radiologic signs, and adjuvant treatment.

**Histopathological diagnosis**	**Entire cohort (*n* = 95)**	**Radiology**				**Treatment**	
		**Edema** **(*n* = 42)**	**Contrast** **(*n* = 90)**	**Cystic** **(*n* = 34)**	**Syrinx** **(*n* = 20)**	**Radiation** **(*n* = 10)**	**Chemotherapy** **(*n* = 6)**
Astrocytoma	5 (5%)	2	5	2	0		
*WHO grade I*	4					1	1
*WHO grade III*	1					1	1
Ependymoma	69 (73%)						
Intramedullary ependymoma	25 (26%)	19	25	14	11		
*WHO grade II*	24					2	–
*WHO grade III*	1					–	1
Juxtamedullary ependymoma	17 (18%)	3	16	3	1		
*WHO grade II*	17					2	–
Myxopapillary ependymoma	27 (28%)	4	25	1	1		
*WHO grade I*	26					–	–
*WHO grade II*	1					–	–
Hemangioblastoma	11 (12%)	8	10	8	4	–	–
Other	10 (11%)	6	9	6	3		
Histology inconclusive	3					2	–
Dermoid	1					–	–
Lymphoma	1					–	1
Lipoma	1					–	–
Melanocytoma	1					–	–
Adenocarcinoma (metastasis)	1					1	1
Subependymoma	1					–	–
PNET	1					1	1

*WHO, World Health Organization; PNET, Primitive neuroectodermal tumor. Variables are presented as count (%)*.

The most common histopathological diagnosis was ependymoma (73%, *n* = 69). We identified 27 myxopapillary ependymomas. Twenty-six of these were WHO grade I, while one showed focal transition into WHO grade II. Excluding the myxopapillary subgroup, the ependymomas composed 62% (*n* = 42) of the remaining IMSCTs. In this cohort 25 were strictly intramedullary ependymomas while 17 of the ependymomas were reported by the surgeon as located juxtamedullary on the surface of the medulla, rather than strictly intramedullary. However, the histopathological analysis of these tumors showed all of these to be typical ependymoma grade II. Two of them were found to be drop metastases from an intracranial tumor, however the other 15 did not show any signs of tumor manifestation elsewhere in the CNS. The subsequent most common diagnoses were hemangioblastoma (12%, *n* = 11) and astrocytoma (5%, *n* = 5) (Table 3).

### Surgical Treatment and Outcome

The median follow-up time was 71 (12–178) months. During this time there were eight deaths, of which three were due to the underlying tumor (metastatic adenocarcinoma, PNET and anaplastic ependymoma). The median time from surgery to death, for these 3 cases, was 19 (19–71) months.

Based on the 3-month postoperative MRI, gross total resection (GTR) was achieved in 81% (*n* = 77) of patients (Table 4). In all cases where the surgeon estimated a GTR (*n* = 72) this was confirmed by the postoperative MRI. Five patients, whose tumors were intraoperatively deemed as only partially resected, showed no remaining tumor on their 3-month postoperative MRI. Four patients underwent biopsy only, with no intention of surgical resection. Intraoperative neurophysiological monitoring was used in 52% (*n* = 49) of all cases, and in 80% (*n* = 40) of the strictly intramedullary tumors. The remaining 10 patients with intramedullary tumors had no IONM due to acute symptom progression require emergency surgery (*n* = 4), only biopsy performed (*n* = 2), surgery performed before IONM was available at our center (*n* = 2), and non-infiltrative hemangioblastoma (*n* = 2). Of the monitored patients, with intramedullary tumors, a permanent intraoperative loss of SEP or MEP was seen in 73% (*n* = 22) and 37% (*n* = 11), respectively, (Table 2).

**Table 4:** Treatment outcome.

**Variable**	**Entire cohort (*n* = 95)**
Duration of follow-up (months)	71 (12–178)
No remaining tumor on post-operative MRI (gross total resection)	77 (81%)
*Tumor recurrence (local)*	0 (0%)
*Tumor recurrence (metastasis)*	3 (3%)
*Disease-free survival (months)*	48 (39–130)
Remaining tumor on post-operative MRI (excluding biopsies)	14 (15%)
*Tumor progression (local)*	7 (7%)
Progression-free survival (months)	22 (8–103)
Re-operation	20 (21%)
***Surgical site***	
*Tethered spinal cord*	2
*Kyphosis*	1
*Wound revision (Infection)*	2
*Wound revision (CSF leak)*	5
***Other site***	
*Herniated disk*	4
***Tumor resection (spinal metastasis)***	
*Hemangioblastoma*	3
*Ependymoma (myxopapillary)*	1
*Ependymoma (juxtamedullary)*	1
*PNET*	1
Death during follow-up	8 (8%)
*Death due to tumor*	3 (3%)
Time to death	72.5 (16–115)

*MRI, magnetic resonance image; PNET, Primitive neuroectodermal tumor. Variables are presented as count (%)*.

In the 77 patients with GTR there were no cases of local tumor recurrence at the primary surgical level, but three cases of intraspinal tumor recurrence at new locations (two grade I myxopapillary ependymomas and one grade II juxtamedullary ependymoma). The median disease-free survival time for these three patients was 48 (39–130) months. One patient with GTR received adjuvant radiotherapy and none received chemotherapy (Supplementary Table 1). The other 74 patients remained tumor free after a median follow up time of 76.5 (12–178) months.

Fourteen patients had remaining tumor on the 3-month post-operative MRI (excluding biopsies). Of these, five patients showed local tumor progression at follow-up and an additional two patients had combined local progression and tumor recurrence at new location. For these seven patients, the median progression-free survival rate was 22 (8–103) months. There were no cases of re-operation due to local tumor progression in the partial resection cohort. Five patients with initial partial resection were treated with adjuvant radiation, one was treated with chemotherapy, and three were treated with combined radiation and chemotherapy. Five patients, all with grade 1 tumors, did not receive any adjuvant therapy. Two of these patients had local tumor progression at follow-up (Table 3, Supplementary Table 1).

For the myxopapillary ependymomas, GTR was achieved in 26 of the 27 patients. As mentioned above, two of these had a ruptured tumor capsule at index surgery, and later showed intradural tumor recurrence at a distant location. In one patient (WHO grade 1), where disseminated tumor spread was identified already at initial diagnosis, only a partial resection was achieved. This patient showed local tumor progression at follow-up.

Of the four patients who only had an initial biopsy one received radiotherapy and two received chemotherapy. Overall, 11% (*n* = 10) of all patients were treated with radiation and 6% (*n*=6) with chemotherapy (Table 3, Supplementary Table 1). In total, 20 (21%) patients required additional spinal surgery during the follow-up period. The reasons were wound revision (*n* = 7; 5 for CFS leak, and 2 for local infection), tumor resection at another site (*n* = 6), herniated disc at an unassociated spinal level (*n* = 4), tethered cord at primary surgical site (*n* = 2), and kyphosis at primary surgical level (*n* = 1) (Table 4).

### Functional Outcome

**Figure 3:**
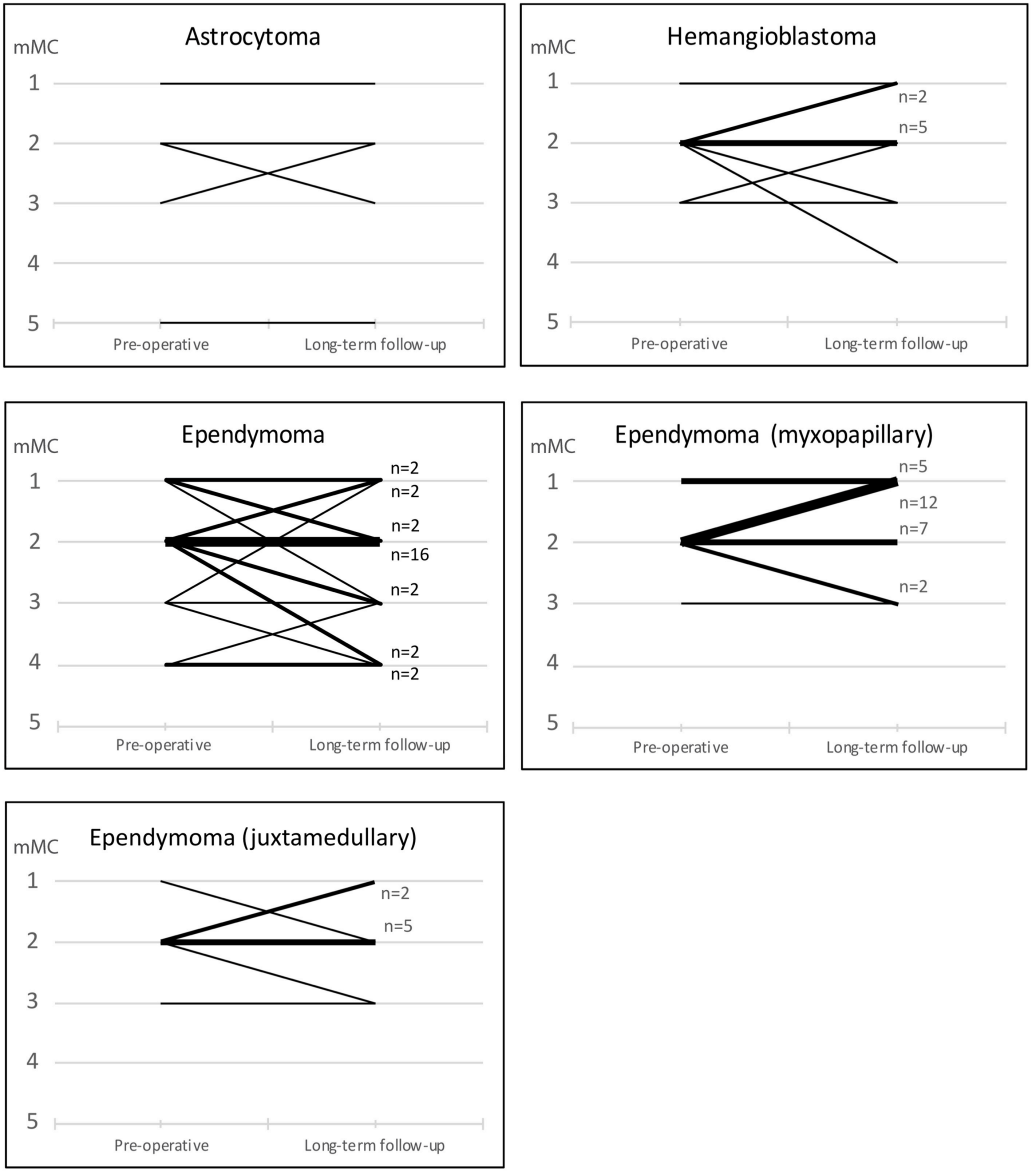
Functional status according to mMCs preoperatively and at long-term follow up. Narrow lines represent individual patients, while bold lines represent groups of patients with similar functional development.

Long term follow-up was defined as the last available clinical record, at least 12 months postoperatively. On an individual basis, 22 patients (23%) showed improvement in mMCs while 20 (21%) deteriorated (Figures 2, 3). On a group level, there was a significant decrease in the share of patients reporting pain (*p* = 0.005) compared to pre-operative values. A decrease in prevalence of sensory deficits was borderline significant (*p* = 0.051). The same pattern was not observed for motor deficits, bladder or gastrointestinal dysfunction (Table 5, Figure 1).

**Table 5:** Functional outcome.

**Variable**	**Pre-operative** **(*n* = 95)**	**Follow-up** **(*n* = 95)**	***p*-value**
Motor deficit	41 (43%)	38 (42%, 5 missing)	0.898
Sensory deficit	60 (63%)	44 (49%, 5 missing)	0.051
Patient reported pain	68 (72%)	46 (50%, 6 missing)	**0.005**
Decreased bladder function	23 (24%)	20 (22%, 5 missing)	0.749
Decreased gastrointestinal function	14 (15%)	7 (8%, 6 missing)	0.143

*Bold text in the p-value column indicates a statistically significant correlation (p < 0.05). MRI, magnetic resonance image. Variables are presented as count (%) or medians (range)*.

Logistic regression was performed to determine risk factors for decreased functional outcome (measured by mMCs) following tumor resection. The four patients that only underwent biopsies were excluded from the analysis. The simple logistic regression showed significance only for subtotal tumor resection (*p* = 0.025). Factors showing a tendency for significance (*p* < 0.1) in the simple logistic regression were put into the multiple logistic regression together with demographic data. The multiple logistic regression yielded significant association of both postoperative tumor remnant (*p* = 0.021) and preoperative functional status (*p* = 0.026) to long term deterioration of functional status (Table 6). Similar results were received when analyzing for deterioration in ASIA IS or for any deterioration including functional scores and urinary or gastrointestinal symptoms (data not shown). Permanent intraoperative loss of MEP/SEP did not show any significant association to long term functional outcome in patients with intramedullary tumor (Table 7).

**Table 6:** Predictors of decreased long-term functional outcome following resection of intra- and juxtamedullary spinal tumors.

**Variable (all included patients)**	**Decreased mMCs (*n* = 18)**	**Not decreased mMCs (*n* = 73)**	**Simple *p*-value**	**Multiple *p*-value**
Age (years)	45.5 (19–72)	43 (16–74)	0.733	0.315
Male gender	12 (67%)	42 (58%)	0.481	0.509
Pre-operative mMCs	2 (1–3)	2 (1–5)	0.062	**0.026**
Pre-operative ASIA IS	D (E–D)	D (E–B)	0.158	–
Intramedullary tumor	12 (67%)	34 (47%)	0.124	–
Cervical engagement	7 (39%)	23 (32%)	0.552	–
Symptom duration (months)	8.5 (0–264)	12 (0–372)	0.623	–
Post-operative MRI rest	6 (33%)	8 (11%)	**0.025**	**0.021**
Re-operation	6 (33%)	13 (18%)	0.154	–

*Bold text in the p-value column indicates a statistically significant correlation (p < 0.05)*.

**Table 7:** Neurophysiological predictors of decreased long-term functional outcome following resection of intramedullary spinal tumors (excluding patients w/o monitoring).

**Variable**	**Decreased mMCs (*n* = 11)**	**Not decreased mMCs (*n* = 27)**	**Simple *p*-value**
Permanent loss of SEP	6 (55%)	15 (55%)	0.465
Permanent loss of MEP	5 (46%)	6 (22%)	0.084
Permanent loss of MEP or SEP	10 (71%)	15 (45%)	0.110

*Variables are presented as count (%) or medians (range). Bold text in the p value column indicates a statistically significant correlation (p < 0.05). mMCs, modified McCormick scale; ASIA IS, American Spinal Injury Association impairment scale; MRI, magnetic resonance image; SEP, somatosensory evoked potentials; MEP, motor evoked potentials*.

## Discussion

Intramedullary spinal cord tumors represent only 3% of all CNS tumors and high-level evidence regarding the treatment of these tumors is lacking. There are no published randomized trials, and to our knowledge there is only one systematic meta-analysis (26). We here present a retrospective cohort study of the demographics, radiological signs, symptoms, and treatment outcomes of these tumors in adults from a single institutions experience. A major strength in this data set is the regional organization of the health care system with a single tertiary referral center within a defined geographic region, ensuring that these data are truly population-based numbers regarding biopsied or operated patients, without referral bias. Corroborating this claim, our data was cross-referenced with the national cancer registry without finding any missed patients. As has been pointed out, population data might otherwise easily be subject to distortion, e.g., due to neurosurgeons' interests or local referral patterns (6, 27).

Radiotherapy has been recommended for ependymomas when GTR cannot be achieved or for disseminated or anaplastic disease (28, 29). According to a review of 348 patients by Oh et al. (30), improved overall survival was only associated with GTR while radiotherapy resulted in prolonged PFS after subtotal resection (STR). Radiation dose however, was not a significant variable. Meanwhile Lee et al. demonstrated that radiotherapy after STR did not correlate with PFS (31). Byun et al reported on a radiotherapy cohort of 25 ependymomas where 68% of the patients experienced acute grade I or II toxicity (28). Sun et al reported that Patients who were treated with adjuvant therapy had a higher risk of progression than those without adjuvant therapy (32). Systematic literature reviews concerning primary IMSCTs show very weak evidence regarding radiation and chemotherapy (1), and there is no standardized protocol for postoperative radiotherapy in IMSCTs. Our policy to avoid radiotherapy for benign and low-grade lesions is supported by our results, no local recurrence was observed when GTR was achieved. In our opinion, post-operative radiotherapy should be reserved for patients with high grade malignancies (WHO grade III-IV) and metastases. Partially resected diffuse astrocytomas are typically managed with serial MRI-evaluations. Some authors suggest postoperative radiotherapy to prevent growth of the residual tumor (33–35), however, this is not supported by our data. In addition, advanced stereotactic delivery systems, such as Cyberknife, are preferred to conventional radiotherapy to decrease the risk of radiation induced myelopathy (36).

It has been argued that the presence of a clear resection plane regardless of tumor histology can better predict whether a GTR is possible (7). However, GTR is more commonly achieved in ependymomas and hemangioblastomas than in astrocytomas (14, 37). Thus, degree of resection, well-defined resection plane, and histopathological diagnosis have been shown to be the most important predictors of recurrence-free survival (8, 14). The impact of GTR on astrocytomas are somewhat controversial with studies reporting both tendencies toward decreased survival (15, 38) and increased survival (34, 39). Since our study includes only 5 patients with astrocytomas, our material is too limited to draw any conclusions in this regard.

Of note, no patient in our study with GTR showed local tumor relapse in the long-term follow-up. Five patients, 3 with GTR, and 2 with partial resection on primary surgery—had intraspinal tumor relapse in other locations than the primary site. Of the 14 patients with remaining tumor on the 3-month postoperative MRI, seven showed local tumor progression on follow-up radiology. Although it should be kept in mind that the partial resection group includes most of the more aggressive tumor entities, this still points to the importance of surgical radicality for long-term tumor control (40).

Acute decline of functional status in the early postoperative period is not unusual. Between 10–40% of patients are reported to show some degree of functional deterioration immediately postoperatively or at early follow-up. However, many of these patients will revert to their preoperative baseline status at long term follow up (>6 mo) with persisting decline in only 7–20%, and up to 40% of all patients show improvement of their functional status compared to preoperatively (8, 9, 14, 41, 42). We found a low frequency of postop neurological deterioration at short term follow-up (Figures 1, 2). However, this is likely due to the timing of our short term follow up which was done 3 months postoperatively, since available data on immediate postop status were not sufficiently standardized to allow statistical evaluation. Other studies have reported high frequencies of early functional deterioration when evaluated in-hospital immediately after surgery, with improvements already after 1 month. At long term follow-up we found no significant alteration in functional score compared to pre-operatively. Twenty-three percent of patients show functional improvement while 21% showed functional decline in mMCs. However, we observed a significant improvement regarding patient reported pain. These results are in line with previous publications.

The most important factor for good functional outcome after surgery is the preoperative functional status (8, 26, 41, 43). Other factors that affect functional outcome are tumor size, tumor location, degree of resection, and patient age (44). High grade tumors generally have worse outcome with functional decline in up to 40–60% (34, 43, 45). In our study, we confirmed preoperative functional status and postoperative tumor remnant as independent predictors for long term functional decline. Unexpectedly, we found no significant correlation of functional deterioration to perioperative loss of SEP/MEP.

A possible explanation is that our material might be underpowered to detect a difference. This is strengthened by the fact that loss of MEP, as predictor of functional outcome, had a low *p*-value (0.084), although not statistically significant, even in this limited material. Our policy is to always use IONM for all strictly intramedullary cases. We found that IONM was used in 80% and was missing in 10 cases with intramedullary tumors. This is in line with recently published data on use (46) or non-use of IONM (47). At our institution, one senior neurophysiologist attends all surgeries with IONM. Due to this fact, IONM cannot be provided without previous planning.

Another potential explanation could have been that loss of SEP/MEP would have led to discontinuation of the surgery in time to prevent neurologic deterioration. However, in this case it would be expected to observe a higher frequency of subtotal resections in the group with loss of SEP/MEP than in the group with no loss, which we did not. Our results are in line with a recent review by Rijs et al. (48), indicating that IONM shows high but not perfect sensitivity and specificity (48).

It has been argued that since preoperative neurologic deficits cannot be expected to resolve, and since surgery in most cases can be performed with limited risk of functional decline for well delineated tumors, surgical resection should be offered to these patients early, rather than to delay intervention until development of progressing neurological symptoms (33). This is in line with our institutional policy and supported by the progression-free survival time in our data.

Among the patients included in this study, 27 patients were treated for myxopapillary ependymoma, including one patient with transition toward a WHO grade II-tumor. For the ordinary grade I myxopapillary tumors surgery is often curative and the long-term outcomes are favorable. However, 3 cases had a spontaneous rupture of the tumor capsule at the time of surgery which results in a benign and easily operated pathology becoming a disseminated and recurring problem for the patient without other treatment options than repeated surgery. Our recommendation, in line with previous reports (49), is therefore that all suspected myxopapillary ependymomas should be operated on regardless of symptoms in order to avoid rupture and dissemination of the tumor.

In summary, our data corroborates previous knowledge on IMSCT and emphasizes the feasibility of surgery as a method of choice for benign or low grade IMSCT. For malignant tumors the benefit of resection remains unclear.

## Conclusion

Surgery is the cornerstone for treatment for IMSCT. Surgical outcome is dependent on careful pre-operative planning, surgical technique, and optimal conditions with IONM. Gross total resection (GTR), with minimal post-operative neurological deterioration, is possible in the majority of the cases, especially in the presence of an identifiable resection plane between tumor and healthy spinal cord. Since long-term progression-free survival could be achieved by GTR without additional adjuvant treatment, we emphasize that low-grade tumors should not be subject to radiotherapy.

## Data Availability

The datasets generated for this study are available on request to the corresponding author.

## Ethics Statement

The studies involving human participants were reviewed and approved by Regional Ethical board in Stockholm, dnr: 2016/1708-31/4. Written informed consent for participation was not required for this study in accordance with the national legislation and the institutional requirements.

## Author Contributions

AE-T, EE, OP, and GB contributed conception and design of the study. AF-S, GB, and OP organized the database. AF-S performed the statistical analysis. OP and AF-S wrote the first draft of the manuscript. AE-T, EE, and GB wrote sections of the manuscript. All authors contributed to manuscript revision, read and approved the submitted version.

### Conflict of Interest Statement

The authors declare that the research was conducted in the absence of any commercial or financial relationships that could be construed as a potential conflict of interest.
